# The gut microbiome of COVID-19 recovered patients returns to uninfected status in a minority-dominated United States cohort

**DOI:** 10.1080/19490976.2021.1926840

**Published:** 2021-06-08

**Authors:** Rachel C. Newsome, Josee Gauthier, Maria C. Hernandez, George E. Abraham, Tanya O. Robinson, Haley B. Williams, Meredith Sloan, Anna Owings, Hannah Laird, Taylor Christian, Yilianys Pride, Kenneth J. Wilson, Mohammad Hasan, Adam Parker, Michal Senitko, Sarah C. Glover, Raad Z. Gharaibeh, Christian Jobin

**Affiliations:** a Department of Medicine, University of Florida College of Medicine, Gainesville, FL, USA; b Department of Medicine, University of Mississippi Medical Center, Jackson, MS, USA; c Department of Infectious Diseases and Immunology, University of Florida College of Veterinary Medicine, Gainesville, FL, USA; d Department of Anatomy and Cell Biology, University of Florida College of Medicine, Gainesville, FL, USA

**Keywords:** Human gut microbiota, SARS-CoV-2, 16S rRNA sequencing, clinical study, microbiome, microbiota, COVID-19

## Abstract

To investigate the relationship between intestinal microbiota and SARS-CoV-2-mediated pathogenicity in a United States, majority African American cohort. We prospectively collected fecal samples from 50 SARS-CoV-2 infected patients, 9 SARS-CoV-2 recovered patients, and 34 uninfected subjects seen by the hospital with unrelated respiratory medical conditions (controls). 16S rRNA sequencing and qPCR analysis was performed on fecal DNA/RNA. The fecal microbial composition was found to be significantly different between SARS-CoV-2 patients and controls (PERMANOVA FDR-*P* = .004), independent of antibiotic exposure. *Peptoniphilus, Corynebacterium* and *Campylobacter* were identified as the three most significantly enriched genera in COVID-19 patients compared to controls. Actively infected patients were also found to have a different gut microbiota than recovered patients (PERMANOVA FDR-*P* = .003), and the most enriched genus in infected patients was *Campylobacter*, with *Agathobacter* and *Faecalibacterium* being enriched in the recovered patients. No difference in microbial community structure between recovered patients and uninfected controls was observed, nor a difference in alpha diversity between the three groups. 24 of the 50 COVID-19 patients (48%) tested positive via RT-qPCR for fecal SARS-CoV-2 RNA. A significant difference in gut microbial composition between SARS-CoV-2 positive and negative samples was observed, with *Klebsiella* and *Agathobacter* being enriched in the positive cohort. No significant associations between microbiome composition and disease severity was found. The intestinal microbiota is sensitive to the presence of SARS-CoV-2, with increased relative abundance of genera (*Campylobacter, Klebsiella*) associated with gastrointestinal (GI) disease. Further studies are needed to investigate the functional impact of SARS-CoV-2 on GI health.

## Introduction

Since the first outbreak of severe acute respiratory syndrome coronavirus (SARS-CoV-2) in December 2019, the skyrocketing number of infections and mortality rate worldwide has made the Coronavirus Disease 2019 (COVID-19) a true pandemic. The lack of widespread vaccine availability and limited treatments represents a formidable challenge to medical systems around the globe, which is in part driven by vulnerable populations with preexisting conditions or those above 50 years old. It is clear that in the absence of preventive measures and with a limited therapeutic arsenal, the number of fatalities associated with COVID-19 infection will remain high until vaccines establish protection. Although COVID-19 is thought to spread by respiratory secretions, a number of observations indicate involvement of the gastrointestinal (GI) tract in the pathogenicity. First, SARS-CoV-2 RNA is detected in the feces of ~50% of COVID-19 patients.^1–4^ In addition, a number of studies have reported gastrointestinal symptoms (nausea, vomiting or diarrhea) in some patients having viral RNA or infectious virus present in their feces.^1,4–10^ Moreover, SARS-CoV-2 spike protein receptor-binding domain (RBD) interacts with the host receptor angiotensin-converting enzyme 2 (ACE2), which is expressed by both lung and intestinal epithelial cells (enterocytes).^8^ Interestingly, a study using a Chinese cohort of 133 patients showed that ~22% of them had an initial or follow-up COVID-19-positive sputum or fecal sample paired with a follow-up negative pharyngeal sample.^11^ Subsequent studies have reported a similar phenomenon of there being a negative viral presence in a pharyngeal sample with a positive presence in matched feces.^1,4,6^ A recent finding showed that human intestinal epithelial cells are readily infected by SARS-CoV-2, supporting viral replication and shedding leading to type I and III interferon responses.^12^ Moreover, intestinal enteroids generated from bats, a natural host for SARS-CoV-2, readily support viral replication, giving credential to the concept of the intestine as a reservoir for the virus.^13^ These findings suggest that presence of SARS-CoV-2 in the intestine may perturb intestinal homeostasis and potentially contribute to disease severity and viral spreading.

The intestinal microbiota forms a symbiotic relationship with its host, contributing to energy and nutrient extraction from diets, shaping immune response, maintaining intestinal mucosal barrier integrity, controlling infection through resource utilization and performing key xenobiotic metabolism.^14–16^ Thus, factors that disrupt microbiota function could promote pathological states such as infection susceptibility and dysregulated immune response. Various studies have shown that the intestinal microbiota promotes the infection of a number of viruses including norovirus, rotavirus, retrovirus and poliovirus.^17^ For example, intestinal microbiota depletion with antibiotics prior to poliovirus infection results in decreased infection susceptibility and minimal viral replication in the intestine of mice.^18,19^ Another example is the impact of microbiota on human immunodeficiency virus (HIV) infection. Studies have demonstrated that HIV infection is associated with intestinal dysbiosis characterized by increased *Prevotella* and a reduction in *Bacteroides*.^20–23^ Recent studies suggest that increased *Prevotella* in HIV is a driver for persistent inflammation in the gut leading to mucosal dysfunction and systemic inflammation.^24–26^ One study suggests that a core microbiota could predict COVID-19 severity in healthy subjects.^27^ Another study shows that the composition of the intestinal microbiota in the Chinese cohort is different between COVID-19 infected and un-infected controls, with symptom severity correlating with specific bacterial taxa.^28^ In this study, we investigated the interaction between intestinal microbiota and SARS-CoV-2-mediated pathogenicity in the first minority-dominated United States cohort.

## Results

### Overview of sample collection and study cohort characteristics

No data are available on the interaction between SARS-CoV-2 infection and intestinal microbiome in a North American cohort. To address this question, we conducted a single-institution study at the University of Mississippi Medical Center (UMC), prospectively collecting fecal samples from a total of 93 patients. Feces from 50 SARS-CoV-2 infected patients were collected within 3 days of ICU admission and 9 SARS-CoV-2 recovered patients were collected when these individuals tested negative for the virus. Feces of 34 SARS-CoV-2 non-infected subjects that were also seen by the hospital for unrelated respiratory medical conditions were used as controls. The majority of the total study population was African American (59 patients, or 63%) and the second most abundant race was Caucasian (Table 1), with this diversity being representative of the patient population seen by UMC. The actively infected SARS-CoV-2 cohort had the highest proportion of patients with co-morbidities, with 60% of patients having diabetes and 84% having hypertension. Additionally, 92% of the infected SARS-CoV-2 patients were treated with antibiotics, while the recovered and control groups had 33% and 15% antibiotic-treated, respectively. 20% of infected patients had diarrhea as a symptom, while no patients in the other two cohorts did, however one patient each in the recovered and control cohorts were diagnosed with IBD. The mortality rate from SARS-CoV-2 in the infected cohort was 64%, with patients having an average stay in the ICU of 14 days. Cohort characteristics such as antibiotic treatment status, age and gender were taken into account in all our microbiota analysis (see Analysis of 16S rRNA gene sequences method section).

**Table 1.** **Overview of study cohort characteristics** Table describing the study cohort characteristics of the prospective clinical trial conducted at UMC for each of the three cohorts: COVID-19 infected, COVID-19 recovered and non-COVID. Values are represented in number and percentage or mean and standard deviation. Differences between the groups were tested using two-tailed Wilcoxon Rank sum test (for continuous variables) and Chi-square test (for non-continuous variables)

	COVID		COVID recovered		Non-COVID	
	Number	Percent or SD	Number	Percent or SD	Number	Percent or SD
Number of participants	50	100%	9	100%	34	100%
Age, mean years	62.3	13.4	46.7	16.1	55.0	15.8
Sex						
Male	28	56%	4	44%	14	41%
Female	22	44%	5	56%	20	59%
Race						
Caucasian	11	22%	4	44%	14	41%
Black	35	70%	4	44%	20	59%
Hispanic	1	2%	1	11%	0	0%
Choctaw	3	6%	0	0%	0	0%
BMI, mean	33.6	9.8	31.5	7.6	26.6	7.9
Diabetic	30	60%	2	22%	3	9%
CKD	11	22%	1	11%	0	0%
CHF	6	12%	0	0%	0	0%
Lung Disorder	11	22%	1	11%	0	0%
Hypertension	42	84%	4	44%	9	26%
IBD	0	0%	1	11%	1	3%
qPCR test for fecal COVID RNA						
Positive	24	48%	0	0%	0	0%
Negative	26	52%	9	100%	34	100%
ICU treatment	49	98%	2	22%	1	3%
Length of ICU stay, mean days (±SD)	14.02	8.61	6.8	10.9	9	N/A
PPI treatment	18	36%	3	33%	11	32%
Antibiotic treatment	46	92%	3	33%	5	15%
WHO						
1–4	3	6%	7	78%	33	97%
5–8	47	94%	2	22%	1	3%
Deceased	32	64%	0	0%	0	0%

### COVID patients have a different gut microbiota than non-COVID controls and recovered patients

**Figure 1.**
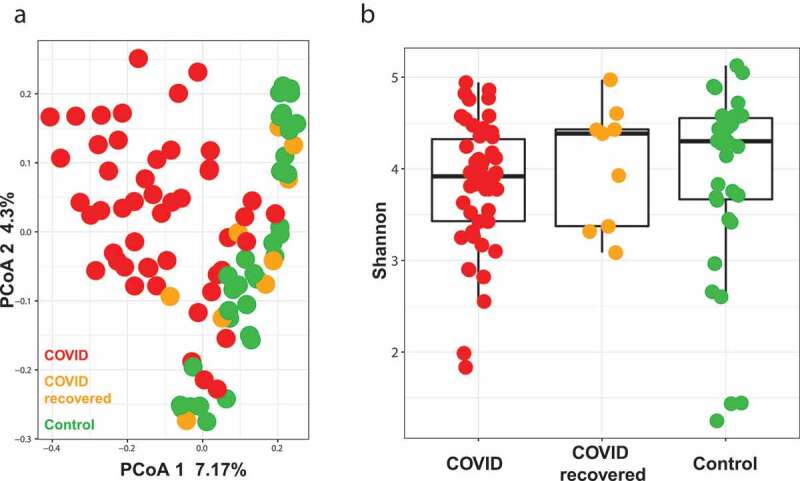
COVID patients have a different gut microbiota than non-infected controls and recovered patients. (a) Principal coordinates analysis (PCoA) showing beta diversity measured by Bray-Curtis dissimilarity between COVID (n = 50), COVID recovered (n = 9) and non-COVID (control, n = 34) subjects. (b) Alpha-diversity as measured by the Shannon index for COVID (n = 50), COVID recovered (n = 9) and non-COVID (control, n = 34) subjects

We examined the fecal microbial composition of the actively infected SARS-CoV-2 patients with that of the recovered and control patients. Broadly, the actively infected SARS-CoV-2 patients were found to cluster separately from both the recovered and non-infected subjects. Importantly, the recovered patients clustered separately from those infected with SARS-CoV-2 and closer to the non-infected control subjects (Figure 1a). The three groups showed similar Shannon diversity index values (Figure 1b), indicating that the separate clustering observed in Figure 1a is not driven by drastic changes in gut microbiota caused by the differing treatment regimens that these patients received (i.e. antibiotic treatment of the COVID patients). Of the two patients in the recovered and control cohorts diagnosed with IBD, neither clustered differently than the non-IBD patients from their respective cohorts.

### COVID patients have a different gut microbiota than non-COVID controls

**Figure 2.**
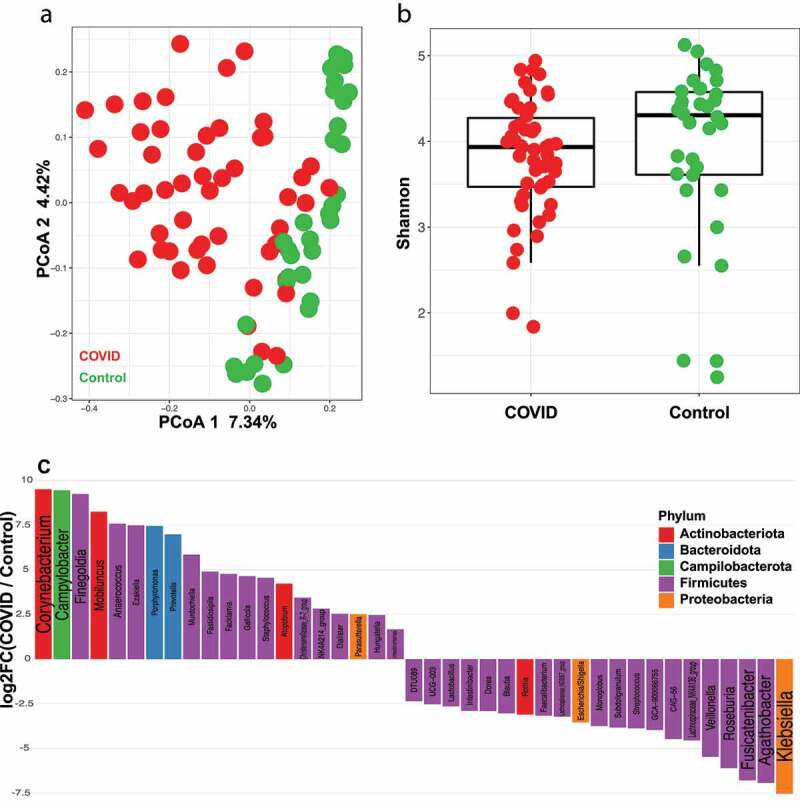
COVID patients have a different gut microbiota than non-infected controls. (a) Principal coordinates analysis (PCoA) showing beta diversity comparison between COVID (n = 50) and non-COVID (control, n = 34) subjects. FDR-*P* = .004 (b) Shannon diversity index for COVID (n = 50) and non-COVID (control, n = 34) subjects. FDR-*P* = .78 (c) Log fold change (logFC) plot of significantly (FDR-*P* < .05) enriched genera in COVID patients and controls. Bars with positive values indicate enrichment in COVID subjects and bars with negative values indicate enrichment in non-COVID subjects. Only the top 75% of significantly enriched genera are shown. See Supplemental Table 2 for full list

When comparing actively infected SARS-CoV-2 patients to control patients, there is a significant difference in gut microbiome (Figure 2a, PERMANOVA FDR-*P* = .004) between the two groups. The difference between SARS-CoV-2 infected and control patients was detected using the antibiotic treated (PERMANOVA FDR-*P* = .002) and antibiotic untreated subjects only (PERMANOVA FDR-*P* = .02) indicating that the detected difference between the two groups is independent of antibiotic exposure. The microbial beta diversity is also affected by both race and gender (Supplementary Table 1) in this cohort, but both do not drive the difference between SARS-CoV-2 and control patients. As proof of concept, analyzing the samples from only African Americans patients show the difference between SARS-CoV-2 and control is significant (PERMANOVA FDR-*P* = .003) but gender is not (PERMANOVA FDR-*P* = .052). We didn’t detect a significant difference between the two groups at the alpha diversity level. Both Shannon diversity index and observed species richness show that both groups have the same level of diversity (Figure 2b, gls FDR-*P* = .78, Supplementary Figure S1A). When examining differences in microbial taxa at the genera level between SARS-CoV-2 and control, *Corynebacterium, Campylobacter* and *Finegoldia* were identified as being the three most significantly enriched genera in COVID patients, while *Klebsiella, Agathobacter* and *Fusicatenibacter* were the top three genera significantly enriched in none SARS-CoV-2 controls (Figure 2c).

### Actively infected COVID-19 patients have a different gut microbiota than recovered patients

**Figure 3.**
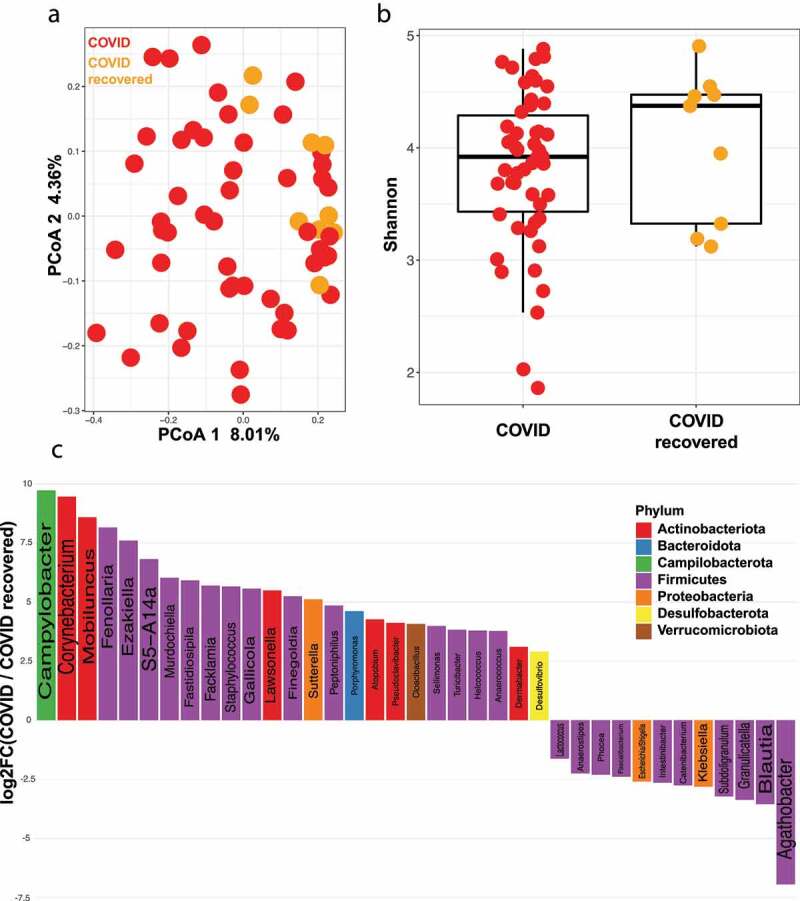
COVID patients have a different gut microbiota than COVID recovered patients. (a) Principal coordinates analysis (PCoA) comparing beta diversity between COVID (n = 50) and COVID recovered (n = 9) subjects. FDR-*P* = .003 (b) Shannon diversity index for COVID (n = 50) and COVID recovered (n = 9) subjects. FDR *P* = .62 (c) Log fold change (logFC) plot of significantly (FDR-*P* < .05) enriched genera in COVID and COVID recovered patients. Bars with positive values indicate enrichment in COVID subjects and bars with negative values indicate enrichment in COVID recovered subjects. Only the top 75% of significantly enriched genera are shown. See Supplemental Table 3 for full list

Nine patients who had recovered from COVID-19 infection gave fecal samples post-recovery, which were compared to those that had been collected from different patients during active infection. Interestingly, there is a significant difference in gut microbiome between COVID-19 patients and recovered COVID-19 patients (Figure 3a, PERMANOVA FDR-*P* = .003) indicating that viral infection is associated with changes in the human gut microbiome that disappear following clearance of the virus. We were also able to detect a significant difference between SARS-CoV-2 infected and recovered patients when using samples from antibiotic untreated subjects only (PERMANOVA FDR-*P* = .04). Again, there is no difference in Shannon diversity index or observed species richness between these two groups (Figure 3b, gls FDR *P* = .62, Supplementary Figure S1B). The most enriched genera in COVID-19 patients was *Campylobacter* (Figure 3c), with the most enriched genus in recovered patients being *Agathobacter* (Figure 3c), which overlaps with the signature found when comparing COVID-19 to the control group. *Blautia* and *Granulicatella* are the second and third most enriched genera in recovered patients, with *Blautia* being associated with overall survival in graft-versus-host disease patients.^29^ Closely following *Blautia* in enrichment in controls is *Faecalibacterium*, an abundant gut commensal that produces short chain fatty acids and is associated with a reduced intestinal inflammation.^30^

### No difference in gut microbiome between recovered COVID-19 patients and non-infected controls

**Figure 4.**
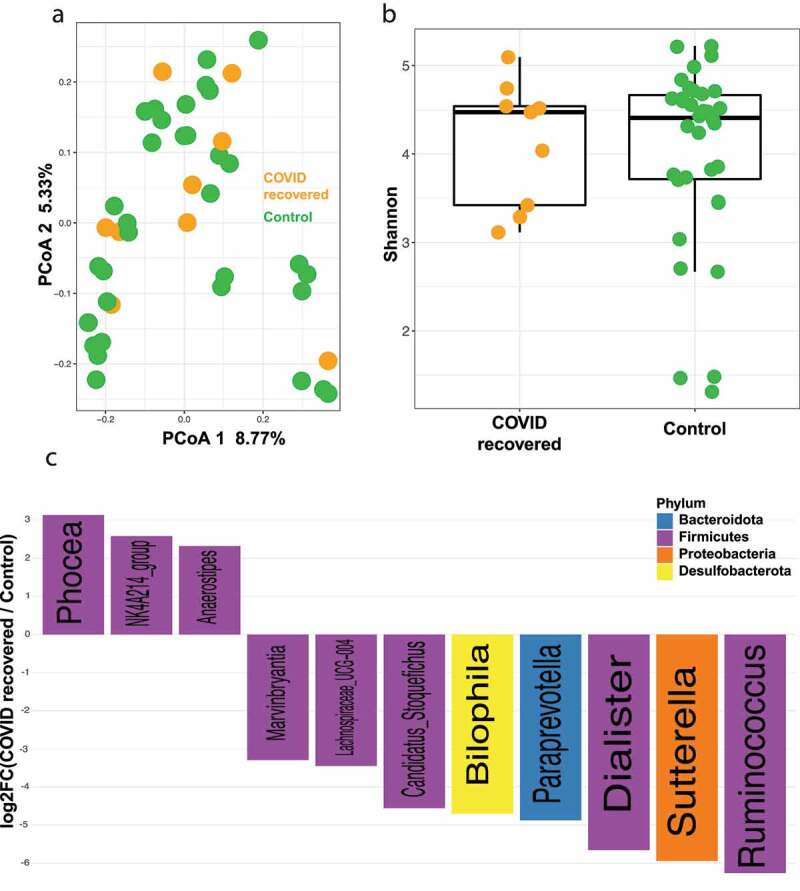
No difference in gut microbiome between recovered COVID-19 patients and non-infected controls. (a) Principal coordinates analysis (PCoA) comparing beta diversity between COVID recovered (n = 9) and control (n = 34) subjects. FDR *P* = .93 (b) Shannon diversity index for COVID recovered (n = 9) and control (n = 34) subjects. FDR *P* = .92 (c) Log fold change (logFC) plot of significantly (FDR-*P* < .05) enriched genera in COVID recovered patients and controls. Bars with positive values indicate enrichment in COVID recovered subjects and bars with negative values indicate enrichment in controls subjects. See Supplemental Table 4 for a list of the plotted genera

To identify the gut microbial changes that occur following infection and recovery from COVID-19, we compared the microbial community structure between recovered patients and controls. There is no difference in gut microbiome between recovered and control patients at both beta (Figure 4a, PERMANOVA FDR *P* = .93) and alpha (Figure 4b, gls FDR *P* = .92, Supplementary Figure S1C) diversity levels. Few taxa were enriched in the recovered vs control groups, with *Phocea* (Figure 4c) being the top genus associated with patients who recovered from COVID-19, and *Akkermansia* being enriched in those who had not been infected (Figure 4c). The similarity between these two cohorts indicates that COVID-19 recovery is also associated with a return of the human gut microbiota to pre-infection community status.

### Presence of detectable virus in feces indicates differences in microbial community structure among COVID-19 infected patients

**Figure 5.**
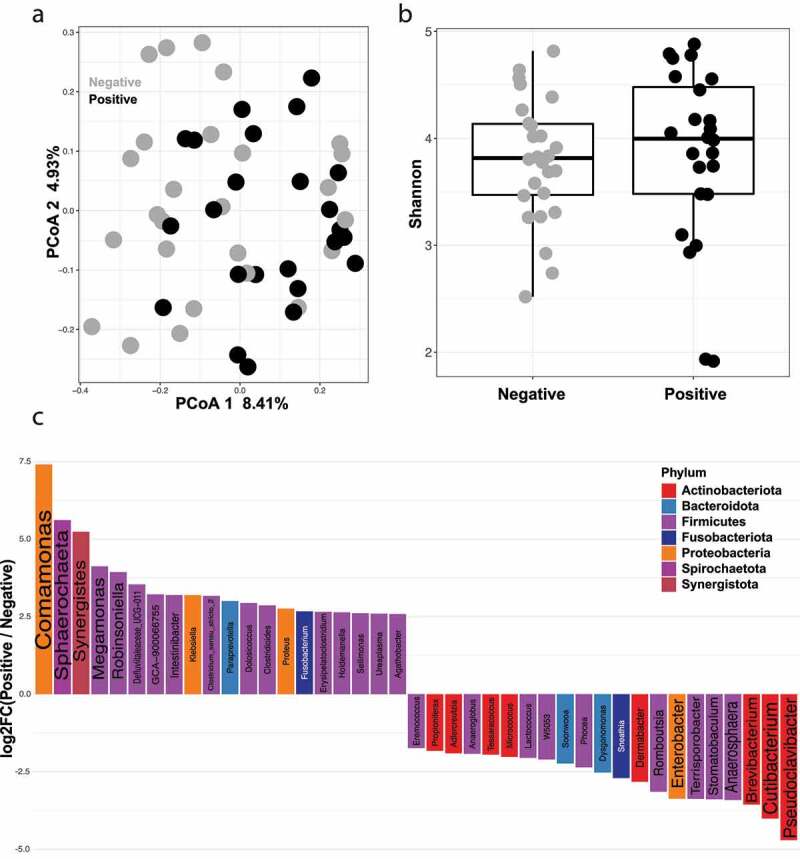
Presence of detectable SARS-CoV-2 virus in feces indicates differences in microbial composition among COVID-19 infected patients. (a) Principal coordinates analysis (PCoA) comparing beta diversity between SARS-CoV-2 qPCR positive (n = 24) and SARS-CoV-2 qPCR negative (n = 26) subjects. FDR-*P* = .03 (b) Shannon diversity index for SARS-CoV-2 qPCR positive (n = 24) and SARS-CoV-2 qPCR negative (n = 26) subjects. FDR *P* = .78 (c) Log fold change (logFC) plot of significantly (FDR-*P* < .05) enriched genera in SARS-CoV-2 qPCR positive and qPCR negative patients. Bars with positive values indicate enrichment in SARS-CoV-2 qPCR positive samples and bars with negative values indicate enrichment in controls SARS-CoV-2 qPCR negative samples. Only the top 75% of significantly enriched genera are shown. See Supplemental Table 5 for full list

To determine if the presence or absence of viral RNA in patient feces results in differences the gut microbiome, each COVID-19 patient’s fecal RNA was isolated and assayed via qPCR for the presence of COVID-19 viral RNA. 24 of the 50 COVID-19 patients (48%) tested positive, and 26 (52%) tested negative (Table 1). We examined the microbial community between qPCR positive and negative patient samples using PERMANOVA and found significant difference between these groups (Figure 5a, PERMANOVA FDR-*P* = .03). This difference was also observed in antibiotic treated subjects only (PERMANOVA FDR-*P* = .02). Consistent with previous observations, the Shannon diversity index or observed species richness between qPCR positive and negative COVID-19 patients is not different (Figure 5b, gls FDR *P* = .78, Supplementary Figure S1D). The microbial taxa enriched in the qPCR positive versus negative samples were then analyzed, revealing that the top three enriched genera in the positive samples were *Comamonas, Sphaerochaeta*, and *Synergistes* (Figure 5c). Also enriched in these positive samples were *Klebsiella* and *Agathobacter*, which were found earlier to be enriched in control and COVID-19-recovered patients compared to COVID-19-infected, respectively. The top three genera enriched in qPCR-negative fecal samples were *Pseudoclavibacter, Cutibacterium* and *Mycoplasma. Phocea* was also enriched in the negative versus positive samples, which was also the genera most associated with COVID-19 recovered patients versus control patients, indicating a possible connection between presence of this bacteria and recovery from COVID-19.

### WHO severity score and Proton Pump Inhibitor (PPI) treatment are not associated with a different gut microbiome within infected patients

The World Health Organization (WHO) disease severity scale for SARS-CoV-2 infection ranges from 1 (asymptomatic) to 8 (death) and is used to assess clinical improvement and survival of SARS-CoV-2 patients over time.^31^ Of the 50 SARS-CoV-2 infected patients, 44 (88%) had a WHO disease severity score between 6–8, indicating hospitalization with severe disease and 6 had a score between 1–5, meaning they were hospitalized with milder disease. 32 patients (64%) received the maximum score of 8, and only 1 patient (2%) received a 3, the lowest score within the infected cohort. When comparing the patients with less severe (score between 1–5) and those with more severe symptoms (between 6–8), no significant difference in gut microbiome was detected (Supplementary Table 1), however this may be due to the majority of patients having very severe symptoms and a high mortality rate. A previous study showed that patients under PPIs medication are at increased risk for severe clinical symptoms of COVID-19.^32^ Thus, we examined the difference in gut microbiome between those patients treated with proton pump inhibitors (PPI) versus those that did not receive them. Eighteen of the infected patients (36%) received PPIs, however PPI treatment didn’t affect the gut microbiome in this cohort (Supplementary Table 1). It has been previously reported that PPI treatment is associated with development of *C. difficile* and other enteric infections and has a significant impact on the gut microbiota, however these findings were taken from the meta-analysis of three cohorts totaling 1815 subjects, and indicates that the dysbiosis seen in our cohort is not due to differences in treatment modalities.^32^

## Discussion

In a United States minority-dominated cohort, we found that COVID-infected subjects have a different gut microbiome composition than both recovered and non-infected groups, and that this altered composition returns to non-infected status upon recovery. Those infected subjects with detectable viral RNA in the feces have a different microbial community structure than those without detectable RNA, indicating a potential impact of SARS-CoV-2 viral infection on bacterial composition and gut health. Importantly, genera enrichment and depletion reported here were independent of the other cohort characteristics such as antibiotic treatment status, age and gender (see Analysis of 16S rRNA gene sequences method section). Previous work has identified the presence of SARS-CoV-2 viral RNA in the feces of 50% of infected patients, pointing to the gut as a potential location of viral replication and infectivity.^33^ Our study using a high-risk cohort falls in line with these previous findings, suggesting that a high number of patients shed SARS-CoV-2 genomic materials through feces. Although the presence of SARS-CoV-2 genetic material could indicate a possible oral-fecal contamination route, studies showing live SARS-CoV-2 in feces are inconclusive.^34,35^

In an exploratory pilot study by Zuo et. al., infected patients in were found to have significantly altered microbial composition compared to both healthy controls and those with non-SARS-CoV-2 respiratory infections.^28^ This dysbiosis was found to persist past respiratory clearance of the virus, regardless of antibiotic treatment, however this was a longitudinal study of a limited pilot cohort of 15 infected subjects from Hong Kong, and comparisons were made to a majority of healthy individuals, not individuals also being hospitalized for unrelated conditions as in our study. Zuo et. al. also found in their seven non-antibiotic treated subjects that there was a significant difference in gut microbial composition associated with disease severity, most notably taxa from the phylum Firmicutes having positive and negative correlation with severity. While we did not find a significant difference in the gut microbiota correlated with disease severity among our 50 infected subjects, one limitation is that 94% of our infected patients had a WHO severity score between 5–8 (severe disease), and 64% percent of these subjects died from their disease, severely underpowering comparisons in severity. Conversely, no infected patients in Zuo et. al.’s cohort died from SARS-CoV-2 and all made a recovery, highlighting a discrepancy in disease severity between the study populations. Clearly, this points to the need for expanded cohorts to identify whether common signal in microbiota differences exist between disease severities. Additionally, although none of the aforementioned studies involved asymptomatic individuals, other groups have examined the differences between those who are asymptomatic and those with moderate to severe symptoms. In a study by Ghoshal et. al., 252 SARS-CoV-2 positive patients, 208 of whom were asymptomatic, were assessed for gastrointestinal symptoms and other factors like co-morbidities, inflammatory bowel diseases, and age.^36^ 25% of symptomatic individuals had gastrointestinal distress and a higher rate of co-morbidities, while those who developed no symptoms had a far lower rate. As of yet there are no published studies examining the gut microbiota between asymptomatic and symptomatic individuals, but a currently recruiting clinical trial in Germany is underway to examine the oral microbiome of approximately 500 asymptomatic subjects (NCT04345510).”

During the preparation of this manuscript a study was published using an expanded cohort of 100 SARS-CoV-2 positive patients from two hospitals in Hong Kong, which showed a difference in gut microbiota composition between COVID-19 infected patients and healthy controls undergoing routine colonoscopy.^37^ The difference in gut microbiota was shown to persist even after the virus was cleared and was also associated with differences in disease severity. It is important to note that critical and severe disease only occurred in 3% and 5% of their COVID-19 infected patients, respectively, as opposed to our cohort in which severe disease was present in 94% of infected subjects. This high rate was a limiting factor in our analysis of microbial composition and disease severity, and Yeoh et. al.’s work raises important questions regarding the long-term consequences of COVID-19 infection in patients recovering from more moderate disease. It is interesting to note that the microbiota of recovered patients returns to non-infected status in our cohort, a contrast with the finding from both Yeoh et. al. and Zuo et. al. This difference may be due to geographical and demographic differences since the latter studies were from the same Hong Kong hospitals compared to our study. Another important difference is that our infected and recovered fecal samples were not taken longitudinally from the same patient. In addition, samples from recovered patients were collected 14 days following COVID-19 negative testing in our study, whereas timeline sampling of recovered specimens from the other studies varied. Although a cross-sectional approach represents a limitation for our study, it must be noted that due to the high disease severity seen in our infected cohort, the small subset of patients that would have survived to give a recovery sample would have prevented comparison analysis. One advantage of our expanded cohort study is the diversity representative of the patient population seen by UMC, and being that African Americans have the highest rate of infection and mortality from SARS-CoV-2, this is reflective of the demographics hardest hit by the SARS-CoV-2 pandemic.^38^ According to a report published by the CDC COVID-19 Response Team, almost all counties in the US identified as being hotspots for infection had a disproportionate number of cases in minority racial and ethnic populations, with the largest disparity existing in Hispanic and African American groups.^39^ These demographic differences highlight the need for expanded multi-center studies to identify the reasons behind the health disparities seen in the COVID-19 pandemic. Additional factors that could potentially contribute to differences between our cohorts and others include diet, lifestyle and co-morbidities, which it must be noted are also associated with differences in gut microbiota composition and health. These demographic differences highlight the need for expanded multi-center studies to identify the reasons behind the health disparities seen in the COVID-19 pandemic. Despite differences in sample size and demographics, both our study and those of Zuo et. al. and Yeoh et al. found an enrichment in *Faecalibacterium* in controls versus infected subjects, pointing to a similar dysbiotic effect of SARS-CoV-2 infection on the gut microbiota. Additionally, our finding of *Campylobacter* enrichment in SARS-CoV-2 infected patients compared to both non-infected controls and recovered patients indicates that this dysbiosis may contribute to the disease pathogenesis. Interestingly, *Campylobacter* is considered to be endemic to the United States, and infection has been shown to result in an asymptomatic carrier state.^40^ The presence of *Campylobacter spp*. is associated with inflammatory bowel disease and development of intestinal inflammation, so the association of SARS-CoV-2 infection with *Campylobacter* presence in the gut microbiota is intriguing.^41,42^ Diarrhea is a frequent symptom of *Campylobacter* infection, and a meta-analysis of 24 cohort or case studies on patients with COVID-19 found that diarrhea frequently presents in these patients (10% of total cases), and recommended the examination of diarrhea as a prognostic factor in SARS-CoV-2 pathogenesis.^43^ In a recent study of 44 COVID-19 infected individuals, higher fecal levels of IL-8, which mediates neutrophil recruitment in inflammation, were found in COVID-19 patients versus controls, while lower levels of IL-10, an anti-inflammatory cytokine, were found in the control subjects. Furthermore, IL-23 fecal levels, which is associated with IBD, were found to correlate with severe disease.^44^ In a study of 990 uninfected subjects, Guo et. al. found that there is a core gut microbiota signature associated with pro-inflammatory cytokines in older, but not younger, individuals. They hypothesize that baseline gut microbial dysbiosis may be a potential driver of the “cytokine storm” seen as the driver of disease severity and mortality COVID-19^27^. Further investigation into the connection between inflammatory response, the gut microbiota and COVID-19 disease severity is warranted. Conversely, the most enriched taxa in our SARS-CoV-2 non-infected cohort is the genus *Klebsiella*, a natural habitant of the large bowel but opportunistic pathogen when colonizing extra-intestinal organs.^45^ Furthermore, *Blautia* and *Faecalibacterium* were found to be enriched in the recovered patients versus infected subjects, and are associated with beneficial fermentation products and short chain fatty acids, respectively, indicating better gut health being associated with recovery.^46^ Further investigation would be needed to determine the functional impact of these biota on GI health using fecal microbiota transplantation.

One confounding factor in our study is the media in which the fecal samples were collected, as most of the COVID-19 patient samples were collected in RNAlater while most of the other samples were immediately frozen without preservative. However, it has been previously shown that preservative media does not significantly affect microbial composition compared to immediate freezing, and a control sample that was collected both in RNAlater and simply frozen showed no difference in composition.^47^ Our cohort includes one recovered subject whose samples were stored in either RNAlater or immediately frozen without preservative. These two samples cluster together in PCoA (Supplementary Figure S2) indicating that the collection method did not contribute to the separate clustering seen in our study. As noted previously, other factors including demography, diet, lifestyle and co-morbidities are far more important when considering the differences between our study and others.

Our findings support the connection between SARS-CoV-2-mediated pathogenesis and the human gut microbiota. The presence of SARS-CoV-2 is associated with an increased relative abundance of *Campylobacter, Klebsiella*, two genera associated with GI disease, while recovered patients displayed a microbiota composition similar to that of un-infected patients. Managing gastrointestinal distress may be key to minimizing the lasting effects of SARS-CoV-2 pathogenesis seen in many patients, and will likely improve the prognosis and outcomes for acutely infected individuals.

## Patients and methods

### Study subjects and design

Eligible patients were recruited from to the University of Mississippi Medical Center (UMMC) medical surgical units, Intensive Care Units (ICU), or endoscopy units between April 2020 and July 2020. The UMMC Institutional Review Board approved the study under IRB#2020-0065. All subjects, or their legally authorized representative provided written informed consent. Subjects were eligible for inclusion in the COVID-19 cohort if they were greater than 18, had a positive nasopharyngeal swab for SARS-CoV-2 by PCR, had COVID 19 related symptoms including fever, chills, cough, shortness of breath, and sore throat and were more than 110 pounds. Subjects were eligible for the recovered COVID cohort if they were greater than age 18, more than 2 weeks post COVID 19 infection that had been confirmed by a positive PCR for SARS-CoV2. Fecal samples were prospectively collected via rectal swab stored in RNAlater from 50 SARS-CoV-2 infected patients within 3 days of ICU admission. Samples from recovered patients were collected either by rectal swab in RNAlater or snap frozen in no preservative at −80 degrees. Under UMMC IRB# 2020–0130, de-identified feces of 34 non-infected subjects also seen by the hospital for unrelated respiratory medical conditions were obtained as controls, which were snap frozen in no preservative at −80 degrees. A limited data set was provided by UMMC center for information and analytics as per this approved, exempt IRB.

### Fecal RNA/DNA extraction and 16S rRNA sequencing

For fecal RNA/DNA extraction, samples were transported from locked storage freezer to BSL-2* Lab. Samples were placed one at a time in the biosafety cabinet to slightly thaw them to allow removal of 50 to 100 ul of stool sample which was placed directly into a 1.7 ml sterile screw cap tube. Lysis buffer (Qiagen: AllPrep® PowerViral® DNA/RNA Kit, Cat. no. 28000–50) was added to the aliquoted fecal sample following manufacturer instruction. Once samples were in lysis buffer, standard BSL-2 procedures were used to extract DNA and RNA according to manufacturer instruction. Following total fecal RNA/DNA extraction, the 16S rRNA V1-V3 hypervariable region was amplified using barcoded primer pairs 27 F (5ʹ-AGAGTTTGATCCTGGCTCAG-3ʹ) and 534 R (5ʹ-ATTACCGCGGCTGCTGG-3ʹ) with universal Illumina paired-end adapter sequences. PCR products were purified, quantified, and pooled as described previously and sequenced in a single run of Illumina MiSeq.^48^

### Analysis of 16S rRNA gene sequences

Demultiplexed reads were imported into DADA2 (v.1.16) pipeline.^49^ Forward reads were used for the downstream analyses and primers from the reads were removed using DADA2 removePrimers function followed by quality filtering and trimming using DADA2 filterAndTrim function with the following options: truncLen = 250, maxN = 0, maxEE = 2, truncQ = 2, rm.phix = T. Sequences were then corrected for Illumina amplicon sequence errors, dereplicated and amplicon sequence variants (ASVs) were generated followed by chimera removal. Taxonomic classification was performed using DADA2 assignTaxonom and addSpecies using silva_nr_v138_train_set.fa.gz and silva_species_assignment_v138.fa.gz, respectively. We then removed any residual sequence that was classified as non-bacterial and all singleton ASVs. This resulted in a total of 5,296,422 reads (56,951 ± 11,958.62 per sample).

We generated Principal Coordinate Analysis (PCoA) using the phyloseq^50^ (v.1.28) R^51^ (v.3.6.3) package from Bray-Curtis dissimilarity matrix after count normalization and log_10_ transformation using the following formula:^52^

$$log_{10}\left(\frac{RC}{n}x\frac{\sum^{x}}{N}+1\right)$$

where *RC* is the read count for a particular ASV in a particular sample, *n* is the total number of reads in that sample, the sum of *x* is the total number of reads in all samples and *N* is the total number of samples.

Alpha diversity (Shannon diversity index) was calculated using the phyloseq R package after rarefying the counts to the minimum count of all samples (1,405 reads). Difference in the microbial community composition (beta diversity) was tested using permutational multivariate analysis of variance (PERMANOVA) through the vegan R package command adonis (v.2.5) with permutations set to 1000. Difference in the microbial community diversity (alpha diversity) was tested using ANOVA on a linear model with generalized least squares (gls) in R nlme package (v.3.1–140). Differential abundance analysis was performed using edgeR^53^ through phyloseq_to_edgeR function at the genus level using ASVs present in at least 10% of the samples. FDR correction was done using R’s p.adjust function employing the method of Benjamini & Hochberg and FDR corrected *P* values < .05 were considered significant.

We first tested if there is a difference in the community using the following model: x ~ var, where x is either Bray-Curtis distance matrix from above or Shannon diversity index and var is status (SARS-CoV-2 infected, SARS-CoV-2 recovered, control subjects) or each of the variables listed in Table 1. Status, antibiotic treatment, race and sex resulted in *P* < .05 and were then used to build a full model in the form of x ~ status + antibiotic treatment + race + gender that was used in the pairwise comparisons. For the SARS-CoV-2 infected cohort, we also evaluated the result of the qPCR test for the presence of fecal COVID RNA, proton pump inhibitors (PPI) usage and WHO severity score using a model of the form: x ~ qPCR result + antibiotic treatment + race + gender + PPI treatment + WHO severity scale.

To confirm that the differences we detected between our comparison groups (Figures 2 and 3) is not due to antibiotic treatment, we performed two additional comparisons in which the difference between SARS-CoV-2 infected and SARS-CoV-2 recovered and SARS-CoV-2 infected and control patients was tested using only the antibiotic treated subjects and then using only the antibiotic untreated subjects.

To rule out the possibility of the enrichment and depletion of genera are due to differences in the cohort characteristics (Table 1), we performed differential abundance tests for each of the variables listed in Table 1 and excluded genera found to be significantly different in any of these tests from Figures 2c, 3c, 4c and 5c.

## RT-PCR

RT-PCR on fecal RNA using two sets of validated primer/probes was performed to establish the presence or absence of SARS-CoV-2 viral RNA. To detect SARS-CoV-2 RNA, we used the Reliance One-Step MultiPlex Supermix (BioRad, cat#12010220) coupled with 2 different sets of primers and probes from IDT (from CDC assays, nCOV_N1 and nCOV_N2, https://www.cdc.gov/coronavirus/2019-ncov/lab/rt-pcr-panel-primer-probes.html). 50 and 150 ng of fecal RNA/DNA was added to each reaction in triplicate, and SARS-CoV-2 RT-PCR was performed as a 1 step procedure using real-time PCR machine (Bio-Rad CFX-384) with appropriate positive and negative controls. Samples were considered positive for COVID-19 RNA if at either RNA concentration the average of the three triplicate Cq values was less than 38. Details of the 16S rRNA gene sequencing analysis are in the supplementary material available online.

## Declarations

### Ethics approval and consent to participate

The UMMC Institutional Review Board approved the study under IRB#2020-0065. All subjects, or their legally authorized representative provided written informed consent. Under UMMC IRB# 2020-0130, de-identified feces of 34 non-infected subjects also seen by the hospital for unrelated respiratory medical conditions were obtained as controls, which were snap frozen in no preservative at −80 degrees. A limited data set was provided by UMMC center for information and analytics as per this approved, exempt IRB.

## Supplementary Material

Supplemental Material

## Data Availability

16S sequencing reads have been deposited in the National Center for Biotechnology Information (NCBI) Sequence Read Archive (SRA) under bioproject ID: PRJNA678695.
