# Supplementary material for: The gut microbiome of COVID-19 recovered patients returns to uninfected status in a minority-dominated United States cohort
Source: Gut Microbes. 2021 Jun 8;13(1):1926840. doi: 10.1080/19490976.2021.1926840 (PMC8205023; doi:10.1080/19490976.2021.1926840)
Supplement: Supplemental Material [file KGMI_A_1926840_SM9016.zip › Supplementary information/Supplementary captions.docx]

**Supplementary Figure 1.** Observed species richness for (A) COVID (n=50) and non-COVID (control, n=34) subjects. (B) COVID (n=50) and COVID recovered (n=9) subjects. (C) COVID recovered (n=9) and control (n=34) subjects. (D) SARS-CoV-2 qPCR positive (n=24) and SARS-CoV-2 qPCR negative (n=26) subjects.

**Supplementary Figure 2.** Principal coordinates analysis (PCoA) comparing beta diversity of RNAlater and flash frozen fecal COVID recovered samples. Subject #256 samples stored in either RNAlater or immediately frozen without preservative cluster together indicating that the collection method does not affect the microbial composition.

**Supplementary Table legends**

Supplementary Table 1. P-values from PERMANOVA (beta diversity) or gls model (alpha diversity) for the significant variables associated with the samples. See methods section for more details.

Supplementary Table 2. significantly (FDR-P < 0.05) enriched genera in COVID patients and controls. Positive logFC values indicate enrichment in COVID subjects and negative values indicate enrichment in control subjects.

Supplementary Table 3. significantly (FDR-P < 0.05) enriched genera in COVID and COVID recovered . Positive logFC values indicate enrichment in COVID subjects and negative values indicate enrichment in COVID recovered subjects.

Supplementary Table 4. significantly (FDR-P < 0.05) enriched genera in COVID recovered patients and controls . Positive logFC values indicate enrichment in COVID recovered subjects and negative values indicate enrichment in control subjects.

Supplementary Table 5. significantly (FDR-P < 0.05) enriched genera in SARS-CoV-2 qPCR positive and qPCR negative. Positive logFC values indicate enrichment in positive subjects and negative values indicate enrichment in negative subjects.
