# Supplementary figures and images for: The gut microbiome of COVID-19 recovered patients returns to uninfected status in a minority-dominated United States cohort
Source: Gut Microbes. 2021 Jun 8;13(1):1926840. doi: 10.1080/19490976.2021.1926840 (PMC8205023; doi:10.1080/19490976.2021.1926840)

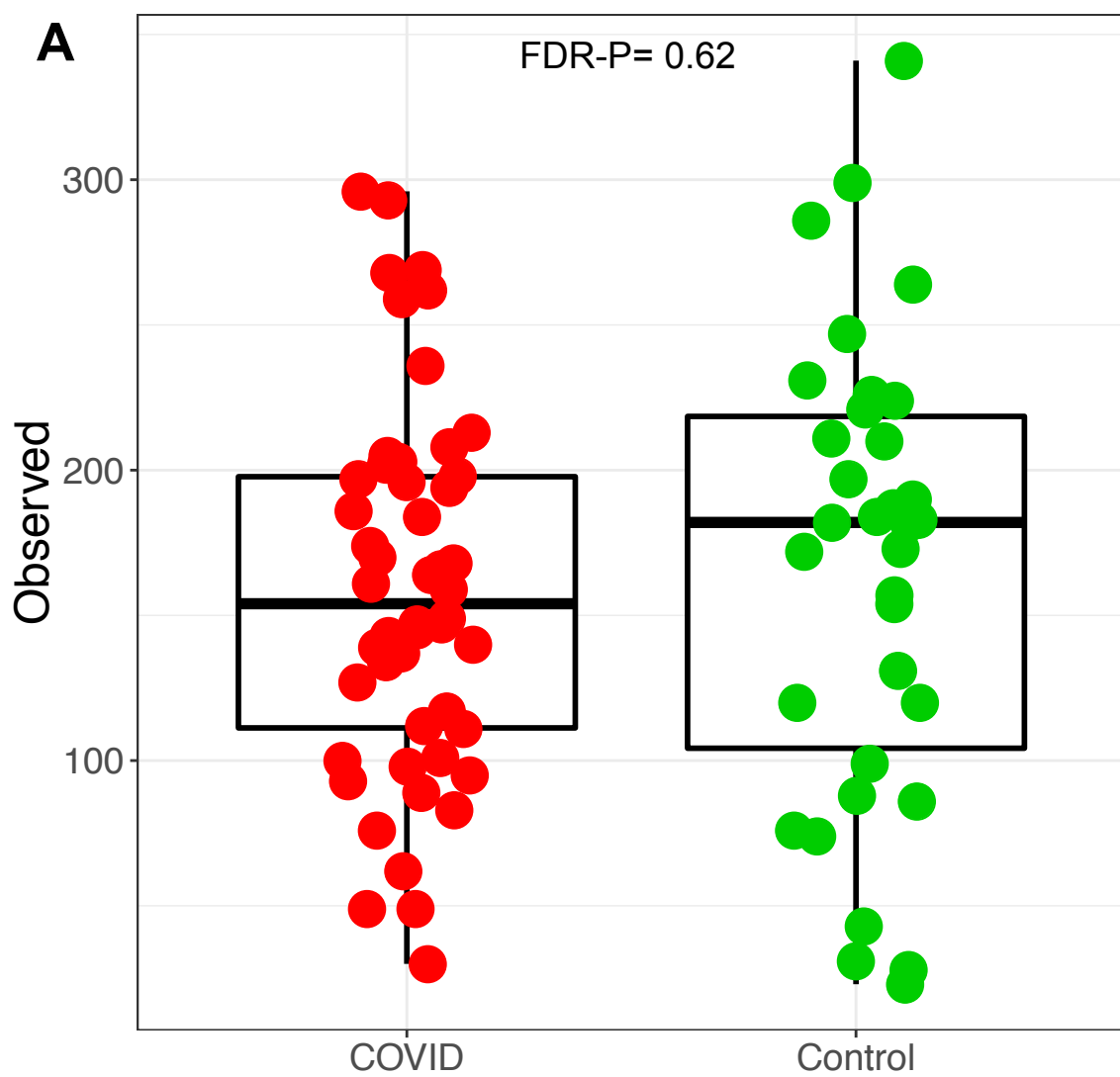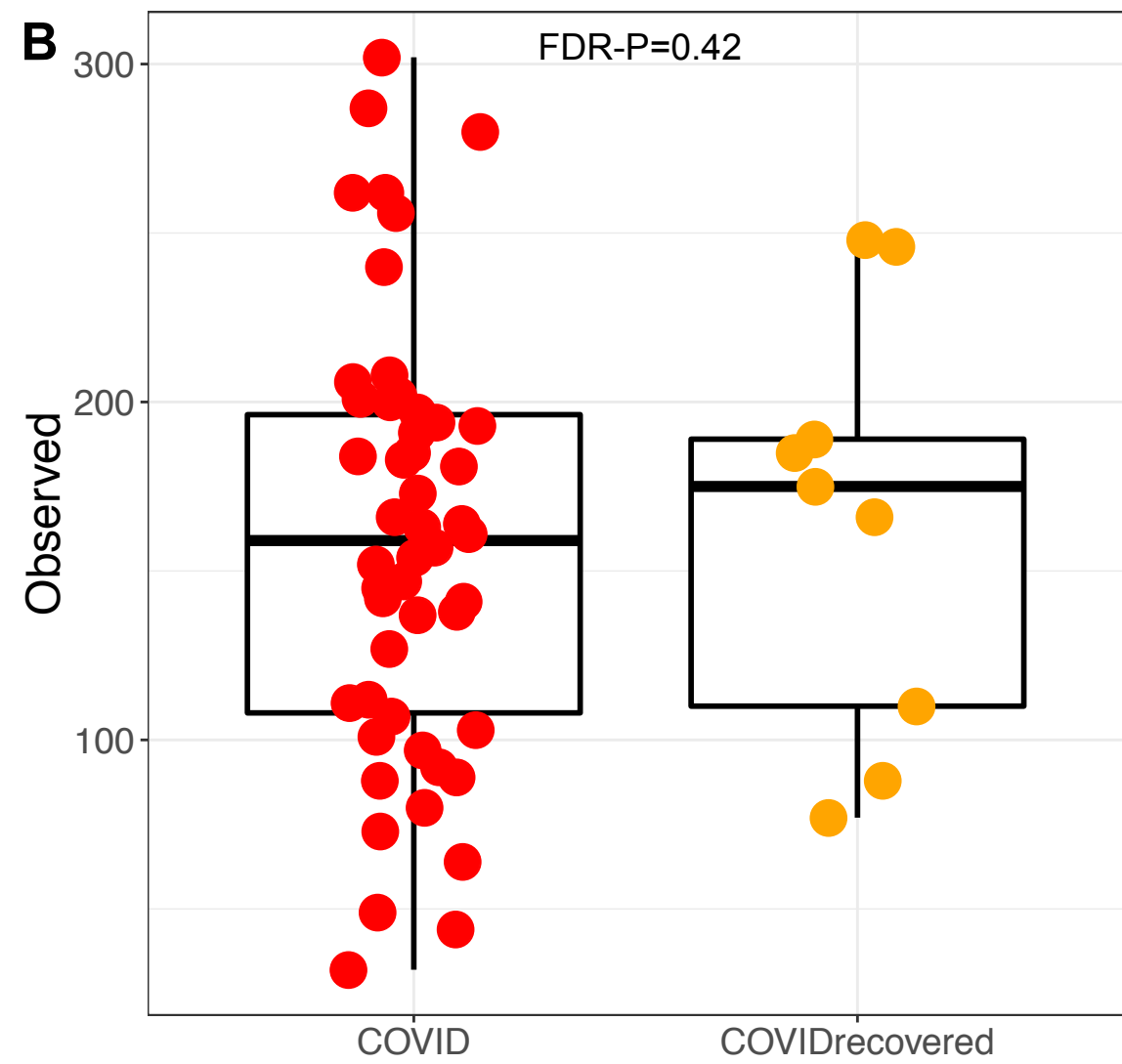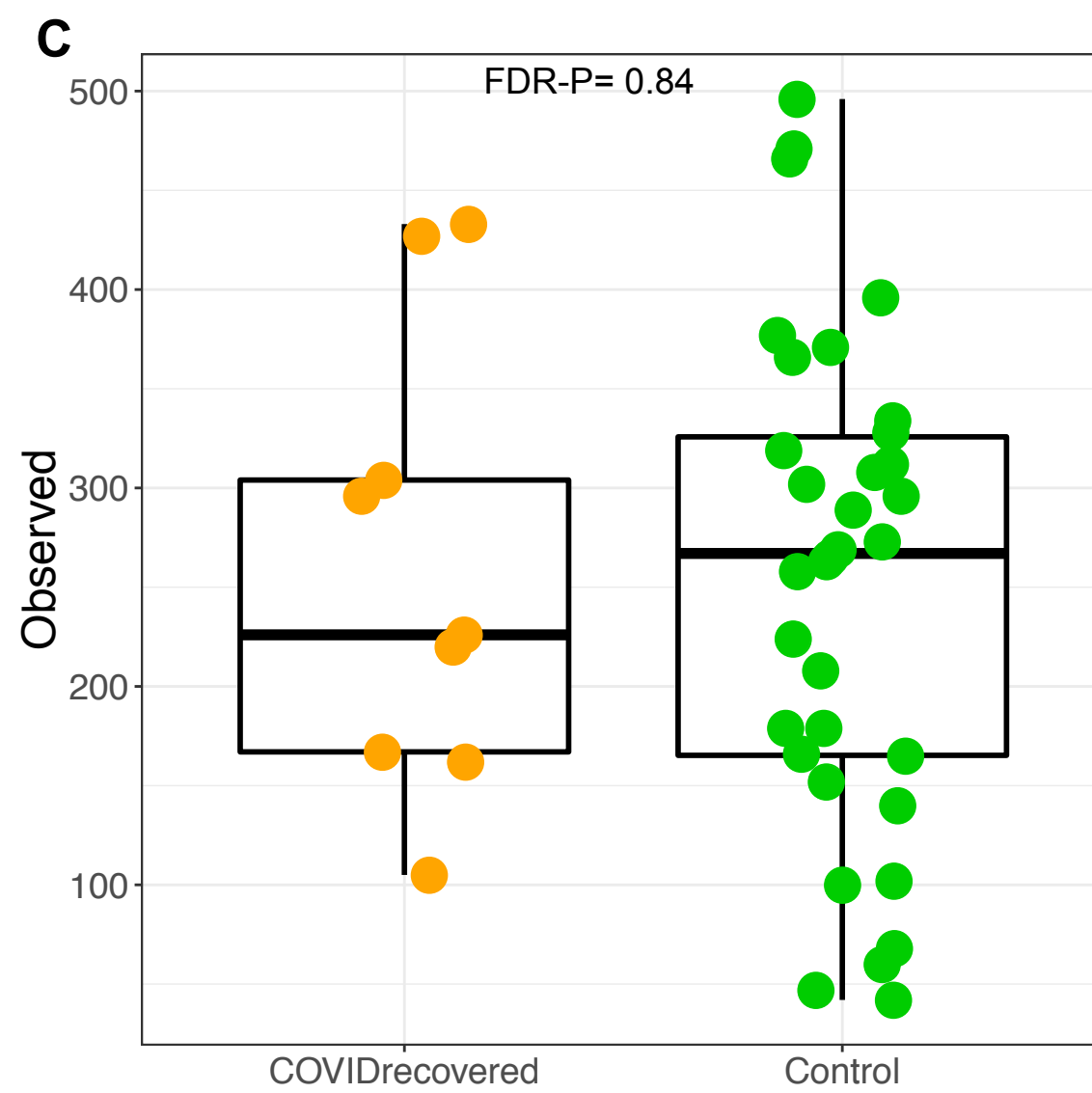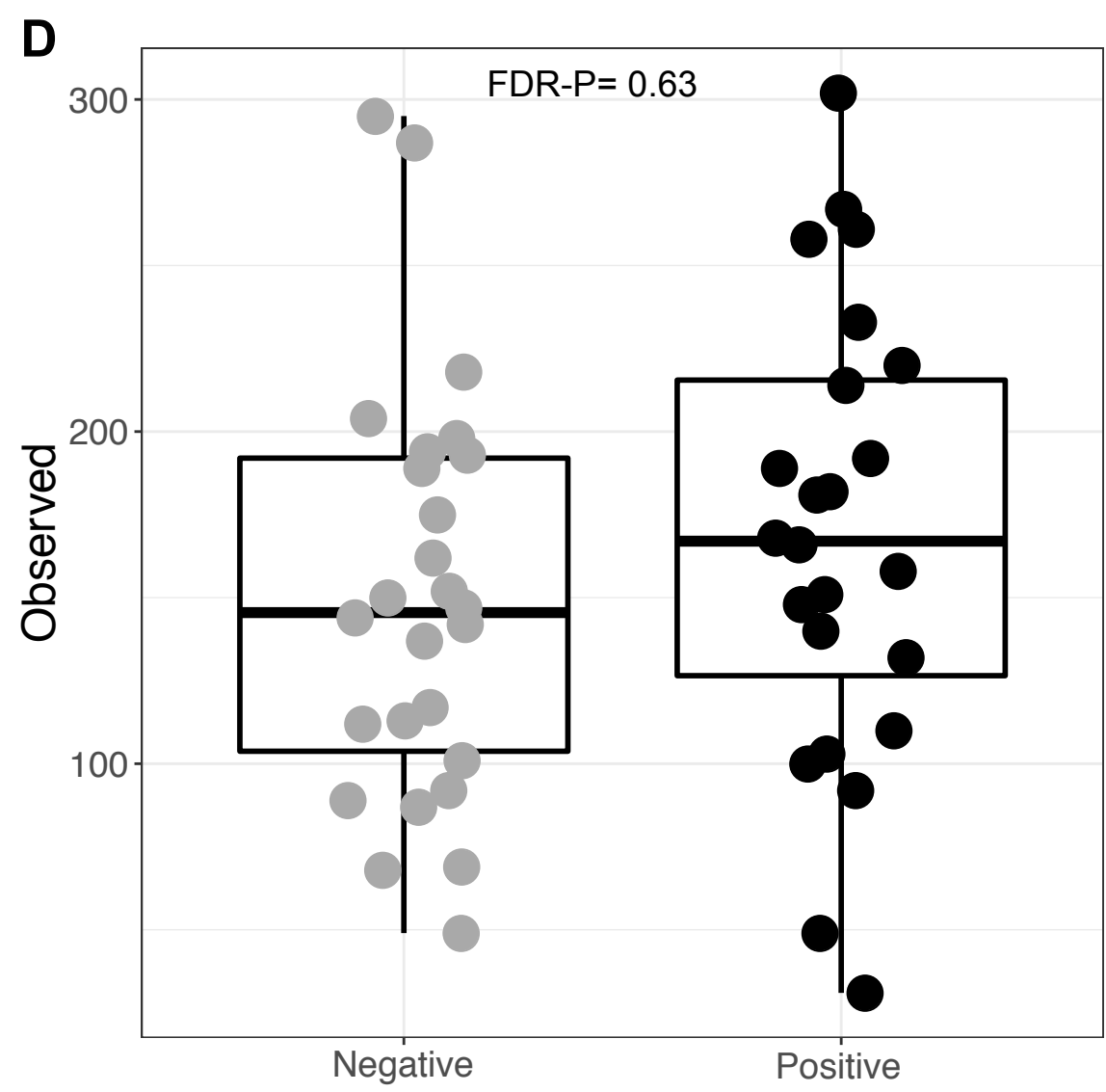

Supplement: Supplemental Material [file KGMI_A_1926840_SM9016.zip › Supplementary information/supplFig01.pdf]

# Figure S1

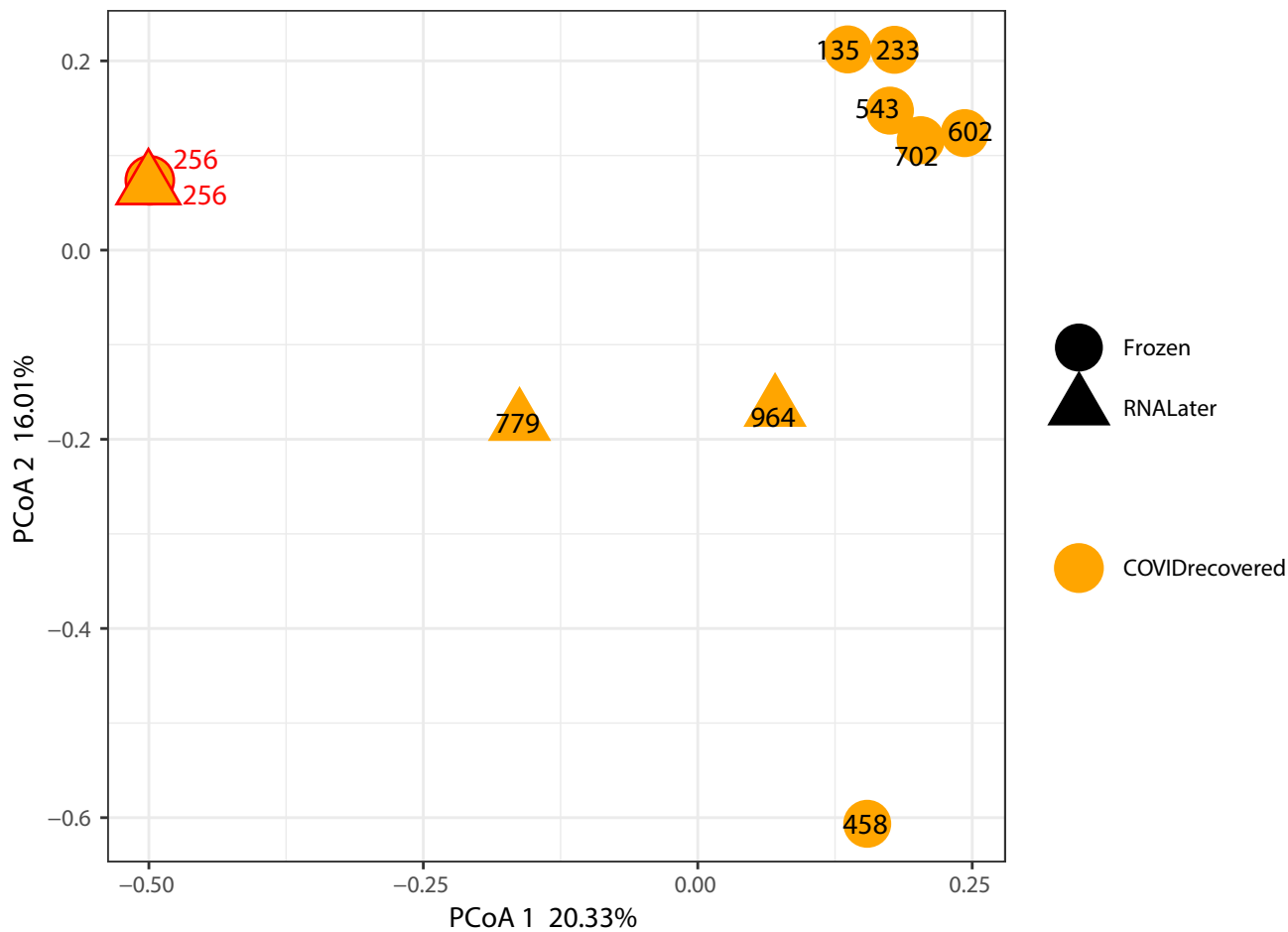

Supplement: Supplemental Material [file KGMI_A_1926840_SM9016.zip › Supplementary information/supplFig02.pdf]
